# Aesthetic Judgments of Live and Recorded Music: Effects of Congruence Between Musical Artist and Piece

**DOI:** 10.3389/fpsyg.2021.618025

**Published:** 2021-02-04

**Authors:** Amy M. Belfi, David W. Samson, Jonathan Crane, Nicholas L. Schmidt

**Affiliations:** ^1^Department of Psychological Science, Missouri University of Science and Technology, Rolla, MO, United States; ^2^Department of Arts, Languages, and Philosophy, Missouri University of Science and Technology, Rolla, MO, United States; ^3^West Point Music Research Center, United States Military Academy, West Point, NY, United States; ^4^Department of Chemical and Biochemical Engineering, Missouri University of Science and Technology, Rolla, MO, United States

**Keywords:** emotion, pleasure, concert, congruity, continuous ratings

## Abstract

The COVID-19 pandemic has brought the live music industry to an abrupt halt; subsequently, musicians are looking for ways to replicate the live concert experience virtually. The present study sought to investigate differences in aesthetic judgments of a live concert vs. a recorded concert, and whether these responses vary based on congruence between musical artist and piece. Participants (*N* = 32) made continuous ratings of their felt pleasure either during a live concert or while viewing an audiovisual recorded version of the same joint concert given by a university band and a United States Army band. Each band played two pieces: a United States patriotic piece (congruent with the army band) and a non-patriotic piece (congruent with the university band). Results indicate that, on average, participants reported more pleasure while listening to pieces that were congruent, which did not vary based on live vs. lab listening context: listeners preferred patriotic music when played by the army band and non-patriotic music when played by the university band. Overall, these results indicate that felt pleasure in response to music may vary based on listener expectations of the musical artist, such that listeners prefer musical pieces that “fit” with the particular artist. When considering implications for concerts during the COVID-19 pandemic, our results indicate that listeners may experience similar degrees of pleasure even while viewing a recorded concert, suggesting that virtual concerts are a reasonable way to elicit pleasure from audiences when live performances are not possible.

## Introduction

Live music has, perhaps until recently, been a growing industry. People are attracted to live music for various reasons, such as wanting to be “a part” of something, sharing the experience with other individuals, and experiencing the novelty of a live event (Brown and Knox, 2017). When asked to recall their favorite musical experiences, most people describe attending live concerts (Krause et al., 2020). While live concerts are a popular aspect of modern culture, the COVID-19 pandemic has brought the live music industry to an abrupt halt. Artists have been looking for ways to replicate the live experience online. However, it is unclear what aspects of a live concert can be communicated virtually and whether listeners could derive a similar amount of pleasure from a virtual event. In the present study, we sought to compare aesthetic judgments (specifically, judgments of experienced pleasure) of a live concert, vs. viewing a recorded version of a concert on a computer. Additionally, the present work sought to investigate whether the musical congruity between artist and piece influences the pleasure a listener experiences, and whether this varies based on the listening setting.

### Listener Responses to Live Music

There is rapidly growing interest in studying cognition and perception in the context of naturalistic settings, such as classrooms (Dikker et al., 2017), movie theaters (Fröber and Thomaschke, 2019), art museums (Brieber et al., 2014; Herrera-Arcos et al., 2017), and live theaters (Ardizzi et al., 2020). Despite this current trend, studies investigating listener responses to music in live concert settings are still relatively uncommon, perhaps due to the logistical challenges and relative lack of experimental control when conducting such work. Although there are difficulties involved in this type of work, it is important to understand the live experience and how it might differ from listening to music in a typical laboratory setting. For example, there are many aspects of the live experience that differ from viewing or listening to recorded music, such as room acoustics and temporal co-presence (Auslander, 2008). Additionally, people choose to attend live concerts so that they can have the shared experience with other audience members, to experience a unique and one-time event, and to be in close proximity with musicians they admire (Brown and Knox, 2017). Live performances therefore may have multiple key dimensions: the “social” aspect of viewing music in the same physical space as other audience members and the performers, and the “liveness” aspect, viewing the performance as it happens in real-time (Swarbrick et al., 2019). In contrast, listeners might prefer some aspects of a recorded concert: For example, the listener is more “in control” of the experience. These are a few of the many dimensions that vary between experiencing live and recorded music.

Some studies of listener responses to live music have taken a broad survey approach, for example, asking listeners to rate their overall impressions of a concert (Thompson, 2006). Using this approach, prior research has indicated that listeners can accurately dissociate their own emotional responses to live music from the emotions expressed in the music itself (Scherer et al., 2019). While this work provides some insight into the live concert experience, one disadvantage is that a summary judgment cannot describe the experience *during* one’s engagement with the music. Conversely, another approach to investigating listener responses to live concerts is to assess listener perceptions and emotions continuously throughout a concert (McAdams, 2004). For example, one study found that continuous ratings of “unexpectedness” and felt emotions during a live concert were associated with quantitative measures of musical expectancy, as determined by a computational model (Egermann et al., 2013). Such studies have also identified relatively high agreement among listeners when making continuous ratings of the “power” of a live performance (Torres-Eliard et al., 2012). Taken together, this work provides additional understanding of the live concert experience and how listener perceptions dynamically unfold during a performance.

Much of the prior research investigating listener responses to live music has focused solely on the live concert experience itself. However, some work has sought to compare listener responses to live music vs. recorded music. In one study, researchers compared listeners’ ratings of a live concert, an audio-visual recording of the concert, an audio-only recording, and a video-only recording (Coutinho and Scherer, 2017). Participants made static, overall ratings of their felt emotions immediately after hearing the music in each of the contexts. The authors found higher interrater reliability in the live concert setting than the other contexts, suggesting higher emotional convergence when viewing a live concert. A similar finding has been identified for continuous ratings of live music: in certain musical settings, continuous ratings were found to show a higher agreement among viewers of live vs. recorded concerts (Upham and Mcadams, 2018). More recently, research has indicated that listeners display more vigorous head movements during a live concert than while listening to a recorded version of the same concert (Swarbrick et al., 2019). Here, we sought to combine both continuous and overall rating approaches, to investigate differences in listeners’ responses to live vs. recorded music.

### Effects of Musical Congruity

In addition to investigating differences between live and recorded music listening, a key component of the present work was to assess whether congruence between musical artist and musical piece influenced listeners’ judgments of music. The idea of musical congruence has been discussed before in the framework of cognitive priming, such that certain musical genres can prime certain semantic concepts (North et al., 2016). Prior work has provided evidence for this by studying the effects of music congruence on purchasing behavior. In one study, participants were given a menu showing food items of different national origins (American, Chinese, or Indian) while listening to either American, Chinese, or Indian music. Results indicated that participants were more likely to choose food items that matched the style of the music (North et al., 2016). There is a substantial literature in the field of consumer behavior and advertising that indicates such music congruence (also called musical “fit”; Herget et al., 2018) influences both choices and aesthetic judgments.

In addition to the work investigating musical congruence in the consumer behavior realm, substantial prior work has indicated cross-modal relationships between music and other sensory stimuli. For example, there are consistent correspondences between musical genres and colors (Palmer et al., 2013; Isbilen and Krumhansl, 2016), tastes (Guetta and Loui, 2017), and styles of movement (Sievers et al., 2013). In sum, this work suggests that music can be perceived as congruent (or incongruent) with other stimuli concurrently present in the environment. In this study, we sought to investigate whether musical congruence influences feelings of pleasure in response to music, by manipulating congruence between the musical artist and the piece of music.

Here, we manipulated musical congruity in terms of the performer and musical piece. Participants viewed performances by two different bands: a university band and a United States army band. Congruity was determined by the style of the musical piece: United States patriotic music was chosen to be congruent with the army band, while non-patriotic music was chosen to be congruent with the university band. For the purposes of this study, non-patriotic music was selected from the standard wind band literature composed by British composers and can be described as “classical” or “concert” music. University bands typically perform as a culmination of their learning particular styles of music and for the overall enjoyment of their community. When United States military bands perform in public settings it is typically for the purposes of demonstrating the professionalism of the armed services and allowing audiences to interact with members of the military in a positive setting. However, it is unclear whether audience members prefer such bands to play traditional United States patriotic music or other styles of music. It is possible that the typical military band attendee already has a pre-determined emotional connection, which is unaffected by the type of music performed. Typical military band attendees may be veterans, have a family member in the armed services, or have a general interest in military music. In addition to investigating the differences between live and recorded music concerts, we sought to discover whether congruence between the band (artist) and music performed influenced pleasure experienced during the performance.

### The Present Study

In the present study, we sought to investigate the degree of pleasure experienced by listeners during live and recorded music, and whether this varied based on the congruence between musical artist and musical piece. We predicted that listeners would experience more pleasure while viewing a live concert than a recorded concert, and that listeners would prefer music that is congruent with the musical artist. Additionally, we sought to explore the possibility of an interaction between musical congruence and liveness – it is possible, for example, that a listener might particularly enjoy seeing their favorite artists play their most well-known song in a live setting; conversely, some evidence indicates that audience members prefer to hear something novel during a live concert (Brown and Knox, 2017). There might be something unique about being present in the room with a musical artist that leads a listener to have stronger expectations about or preferences for what they will perform, and therefore potentially exhibit stronger effects of congruence. In addition to having interesting theoretical implications, we sought to examine this effect as it may have practical implications for the future programming of concerts. For example, artists might select different pieces based on whether the concert would be presented online or performed in front of a live audience.

In the present study, participants either viewed a live performance of a joint concert of the Missouri S&T Bands and the 399th Army Band, or viewed an audiovisual recorded version of the same concert in isolation. Musical congruence was manipulated by programming two pieces for each band: one patriotic piece for each group (i.e., congruent with the Army band) and one non-patriotic piece. To investigate the effects on both continuous and overall ratings, listeners continuously rated their felt pleasure during each of these four pieces, and made overall ratings before the concert, during intermission, and at the conclusion of the concert. Additionally, to address potential effects of piece order and other confounds inherent in the naturalistic concert setting, we conducted a brief follow-up experiment in which online participants listened to and rated the audio-only of the musical pieces, in a randomized order.

## Materials and Methods

### Participants

Thirty-two individuals participated in this study. Participants consisted of two groups: one group completed the experiment in a live concert setting (“Live” condition), and a second group completed the experiment in a lab setting (“Lab” condition). All participants were recruited through advertisements in the local community and were compensated $20 for their participation in the study. All procedures were approved by the Institutional Review Board at Missouri S&T.

To determine an appropriate sample size necessary to test our effects of interest, we conducted a power analysis using G*Power software (Faul et al., 2007). We first identified our expected effect size from a similar study, which tested differences in aesthetic appreciation of viewing a film in a movie theater vs. at home (Fröber and Thomaschke, 2019). This study reported an effect size *η*^2^ = 0.416 for the main effect of viewing condition. Assuming this effect size, we conducted an *a priori* power analysis to determine the sample size necessary to detect a significant main effect of listening condition (live vs. lab), which resulted in a total sample size of 14 (i.e., seven per group). While this power analysis indicated the minimum number of participants sufficient to detect our predicted effect of interest, we also sought to maximize our number of participants. We anticipated some potential data loss, given the challenges associated with recording continuous ratings during a live concert, and wanted to over-recruit due to the lack of possibility for collecting data in the future given the one-time nature of the concert. We were limited in our participant number due to space constraints in the theater – a maximum of 20 persons could be seated in an upper balcony area. As we were conducting this experiment in a “real,” ecologically valid concert setting (as opposed to holding a concert specifically for the purpose of this experiment), we sought to minimize distractions for the public audience attending the concert. Therefore, we recruited 20 participants for the live study based on these factors. As anticipated, there was some participant dropout for certain musical pieces due to technical difficulties and participant error (see Table 1 for *Ns* for each piece).

**Table 1:** Number of participants with continuous data for each piece.

Piece number	Piece name and composer	Band	Piece type	*N* Live	*N* Lab
1	*English Folk Song Suite* (movement 3) by Ralph Vaughan Williams	University	Non-Patriotic	10	10
2	*Washington Post March* by John Philip Sousa	University	Patriotic	12	12
3	*First Suite in E-flat for Military Band* (movement 3) by Gustav Holst	Army	Non-Patriotic	15	12
4	*The Stars and Stripes Forever* by John Philip Sousa	Army	Patriotic	14	12

To match the number of participants with full data in the live condition, we recruited 12 participants in the lab condition. Demographics for both groups are as follows: Participants in the live condition (demographics data only obtained for 12 participants) consisted of seven women and four men. Participants in the live condition were, on average 26.09years old (SD = 12.07), had completed 15.45years of education (SD = 3.26), and had 4.45years of musical training (SD = 4.98). Lab participants (*N* = 12) consisted of four women and seven men, with an average age of 21.84years (SD = 2.70), 15.92years of education (SD = 1.70), and 6.18years of musical training (SD = 5.05). A chi-squared test revealed that there was not a significant difference in the proportion of men and women between the live and lab groups, *X*^2^ = 2.59, *p* = 0.10, 95% CI of the difference in proportions: (−0.70, 0.05). Independent-samples *t*-tests also revealed no significant differences between the groups in age [*t*(22) = −1.23, *p* = 0.22, 95% CI of the difference in means: (−11.36, 2.87)], years of education [*t*(22) = 0.45, *p* = 0.65, 95% CI of the difference in means: (−1.68, 2.62)], or years of musical training [*t*(20) = 0.80, *p* = 0.42, 95% CI of the difference in means: (−2.73, 6.19)]. None of the participants were members of the United States military. Four of the lab participants had family members in the United States military, as did four of the participants in the live setting.

### Stimuli

The “stimulus” in this case was a live concert or a recorded version of the concert. The event chosen was a joint concert of the Missouri S&T Bands (the University Wind Symphony and S&T Symphonic Band) and the 399th Army Band from Fort Leonard Wood. This is an annual event on campus and as such was already suited to accommodate the subjects in the live concert group. Both bands were aware of the study taking place, but with it being a regularly scheduled event it had no bearing on how the bands prepared the musical works.

While participants in the live concert setting were in attendance for the entire concert, they were only asked to rate four of the works performed; two by Missouri S&T’s University Wind Symphony (one patriotic, one non-patriotic) and two by the 399th Army Band (one patriotic, one non-patriotic). To keep the conditions similar between rating the college and military bands, both ensembles performed a three-movement band suite by a British composer in which the final movement was in a march style, followed immediately by an American march by John Philip Sousa. In this way, subjects could rate two contrasting pieces back-to-back. Between the performances of the University Wind Symphony and the 399th Army Band a third ensemble performed a set as a part of the overall concert program. During this portion of the program, participants were given a break.

The non-patriotic piece by the college band (Piece 1) was the *English Folk Song Suite* by Ralph Vaughan Williams (with only the third movement being rated by the subjects); the patriotic piece by the college band (Piece 2) was the *Washington Post March* by John Philip Sousa. The non-patriotic piece by the 399th Army Band (Piece 3) was the *First Suite in E-flat for Military Band* by Gustav Holst (again, with only the third movement being rated by the subjects) and the patriotic piece (Piece 4) was *The Stars and Stripes Forever* by John Philip Sousa. All of the pieces were selected based on the bands’ familiarity with them as well as their relatively short length. The two Sousa marches are of similar style and are familiar to most American audiences. Both of the British suites, on the other hand, are not as well-known to the general public, but other factors including the similar style of their “March” movements (containing both march and lyrical elements) that contrast the marches of Sousa were considered in their selection.

### Procedure

Upon arriving at the concert or the lab, participants completed the informed consent procedures and were instructed on how to use the continuous rating scale. In the live condition, participants were seated in an upper balcony area of the theater. All participants were seated together in the same area, to avoid distracting the rest of the audience. In the lab condition, participants were seated at a computer (21-in iMac) and watched a video recording of the concert on its screen. The audio was played through the computer speakers. Participants in the lab watched the video recording in a private testing room by themselves, although a research assistant was present to answer any questions throughout the experiment. Participants in both conditions completed the same procedure and were given the same instructions. Procedural details are as follows:

#### Overall Ratings

To assess changes in feelings and mood over time, participants completed a brief questionnaire at three-time points: immediately before viewing the concert (“pre-concert”), during intermission (“mid-concert”), and immediately after the concert (“post-concert”). For each question, participants were shown the question text and a visual slider bar with three text anchors. Slider bar ratings ranged from 0 to 100, although participants did not see these numerical values when making their ratings. Questions and anchors were as follows: “In general, what are your feelings towards the United States Military?” (“Very negative,” “Neutral,” “Very positive”); “How likely are you to attend a concert by a United States Military Band in the future?” (“Not at all likely,” “Somewhat likely,” “Very likely”); “In general, what are your feelings about attending live concerts?” (“Very negative,” “Neutral,” “Very positive”); “How likely are you to attend a concert by the Missouri S&T Band in the future?” (“Not at all likely,” “Somewhat likely,” “Very likely”). In addition to these questions, participants also completed the Positive and Negative Affect Schedule (PANAS) at each time point, as a measure of current affect (Watson et al., 1988).

#### Continuous Ratings

Participants continuously rated each of the four chosen pieces of music using the EmotionTracker app (Brielmann and Pelli, 2017; Brielmann et al., 2017). Ratings were made using the touchscreen of the participants’ smartphones. Ratings were made by placing two fingers on the touchscreen; opening the fingers wider indicated that the participants were experiencing more pleasure and closing the fingers closely together indicated that the participants were experiencing less pleasure. Prior to rating the pieces, participants were asked to provide their “maximum” rating by spreading their fingers as wide as comfortably possible, and their “minimum” rating by relaxing their fingers to the minimum spread that was comfortable at rest. Therefore, each participant’s data was scaled based on their individual maximum and minimum, and all ratings were numerically mapped on to these values on a 10-point scale (minimum = 1, maximum = 10). This calibration process was done before each of the four pieces. Following the calibration, participants were asked to continuously rate how much pleasure they were experiencing during the four pieces of music. Finger spread on the touchscreen was sampled at 1Hz.

## Results

### Overall Ratings

First, we sought to assess the overall impact of the performances on listeners’ affective responses (positive and negative affect, measured by the PANAS), feelings towards the United States military, feelings about live concerts in general, and likelihood to attend concerts by army bands and the university bands. For each of these dependent variables, we conducted a separate mixed-ANOVA with time (pre-concert, mid-concert, post-concert) as the within-subjects variable and condition (lab, live) as the between-subjects variable. All analyses were conducted using R (*v*3.6.2). ANOVAs were conducted using the anova_test function from the rstatix package (*v*0.5.0; Kassambara, 2020).

**Figure 1:**
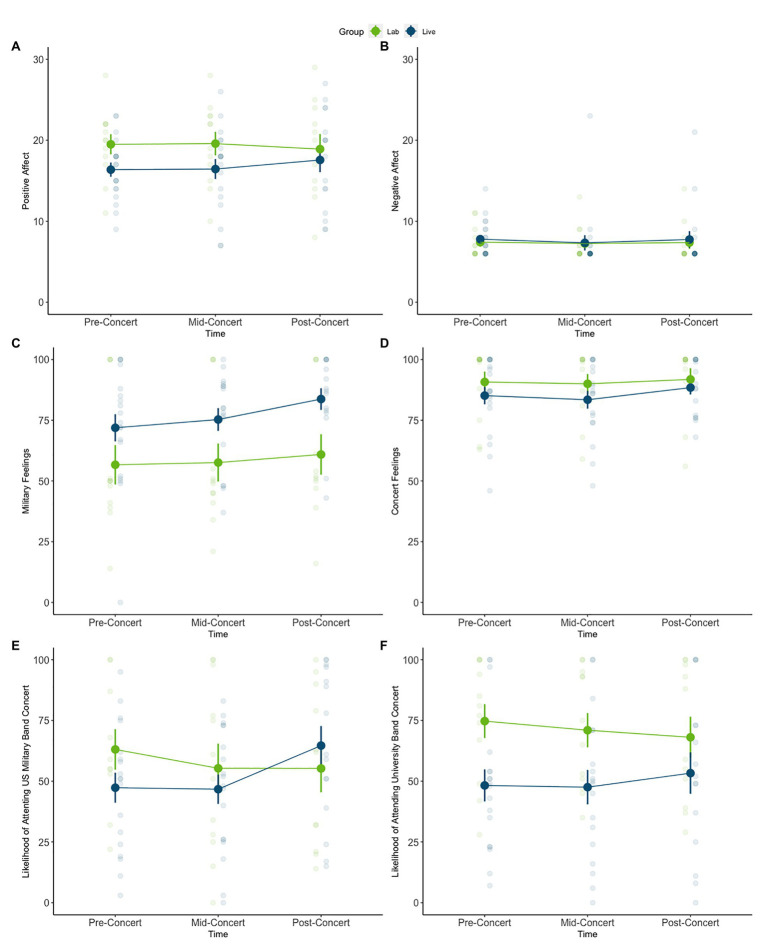
Results from the pre-, mid-, and post-concert ratings. **(A)** Positive affect, **(B)** negative affect, **(C)** feelings about the US military, **(D)** feelings about attending concerts, **(E)** likelihood of attending US military band concerts, and **(F)** likelihood of attending university band concerts. All results are non-significant except for the listeners’ ratings of military feelings. For military feelings, there was a significant main effect of condition (live group rated higher than lab group) and a significant main effect of time (post-concert ratings higher than mid-concert ratings). Opaque circles indicate mean; error bars indicate standard error of the mean; transparent circles indicate individual participant data points.

For PANAS positive affect, there were no significant effects of condition [*F*(1,24) = 1.59, *p* = 0.21, *η_p_^2^* = 0.06], time [*F*(2,48) = 0.10, *p* = 0.89, *η_p_^2^* = 0.004] or interaction between time and condition [*F*(2,48) = 0.84, *p* = 0.43, *η_p_^2^* = 0.03]. Similarly, for PANAS negative affect, there were no significant effects of condition [*F*(1,24) = 0.10, *p* = 0.74, *η_p_^2^* = 0.004], time [*F*(2,48) = 0.13, *p* = 0.87, *η_p_^2^* = 0.006] or interaction between time and condition [*F*(2,48) = 0.01, *p* = 0.98, *η_p_^2^* = 0.0007]. See Figures 1A,B for a graphical depiction of the results.

When assessing listeners’ feelings about the United States military, there was a significant main effect of condition [*F*(1,24) = 5.58, *p* = 0.02, *η_p_^2^* = 0.19] such that overall, the live group reported more positive feelings towards the United States military than the lab group (Figure 1C). There was also a significant main effect of time [*F*(2,48) = 4.74, *p* = 0.01, *η_p_^2^* = 0.17]. *Post hoc* tests, Bonferroni corrected for multiple comparisons, indicated that post-concert ratings were significantly higher than mid-concert ratings (*t* = −3.02, *p* = 0.01). There was no interaction between condition and time [*F*(2,48) = 0.50, *p* = 0.56, *η_p_^2^* = 0.02]. When assessing listeners’ feelings about attending concerts in general, there were no significant effects of condition [*F*(1,24) = 0.45, *p* = 0.50, *η_p_^2^* = 0.01], time [*F*(2,48) = 2.07, *p* = 0.13, *η_p_^2^* = 0.07], or interaction between condition and time [*F*(2,48) = 0.31, *p* = 0.73, *η_p_^2^* = 0.01; Figure 1D].

When assessing the likelihood of attending an army band concert in the future, there were no significant main effects of condition [*F*(1,21) = 0.39, *p* = 0.053, *η_p_^2^* = 0.01], time [*F*(2,42) = 3.79, *p* = 0.051, *η_p_^2^* = 0.15], or interaction between time and condition [*F*(2,42) = 3.35, *p* = 0.06, *η_p_^2^* = 0.13; Figure 1E]. When assessing the likelihood of attending a university band concert in the future, there were also no significant main effects of condition [*F*(1,24) = 4.09, *p* = 0.054, *η_p_^2^* = 0.013], time [*F*(2,48) = 0.24, *p* = 0.81, *η_p_^2^* = 0.0009], or interaction between condition and time [*F*(2,48) = 1.82, *p* = 0.17, *η_p_^2^* = 0.008, Figure 1F].

### Continuous Ratings

Based on prior research (Schafer et al., 2014), we selected three metrics of the continuous ratings to analyze: mean (as a measure of the average pleasure felt during the piece), maximum value (as a measure of the peak amount of pleasure felt during the piece), and standard deviation (as a measure of the variation in pleasure felt). These three metrics (mean, max, and SD) were calculated for each participant, for each piece of music. We then conducted a linear mixed-effects model for each of the three metrics. In this model, condition (live, lab), piece type (patriotic, non-patriotic), and band (army, university), as well as all their interactions, were considered as fixed effects. Categorical predictors were coded as follows: Condition (lab = −0.5, live = 0.5); piece type (non-patriotic = −0.5, patriotic = 0.5); band (army = −0.5, university = 0.5). We included intercepts for participants as random effects. Linear mixed-effects models were performed and corresponding *p*-values were calculated using the lmer function from the lmerTest package in R (Kuznetsova et al., 2017). *Post hoc* pairwise comparisons were calculated using the emmeans function from the emmeans package in R (Lenth, 2020). Due to the fact that not all participants rated every piece, we conducted all analyses reported below a second time, including only those participants who rated every piece; this did not substantially change the results reported here (see Supplementary Material for the details of these additional analyses).

**Figure 2:**
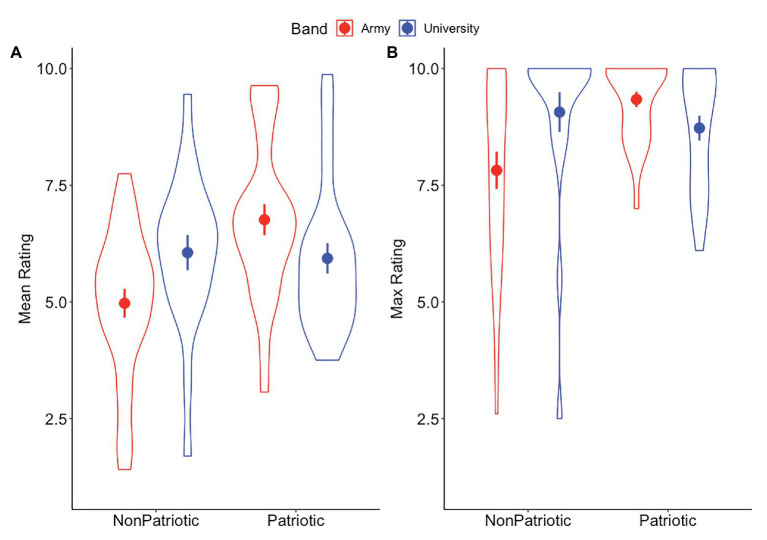
Results from the continuous ratings metrics. Circles indicate means, error bars indicate standard error of the mean. Violin plots illustrate distributions of the individual data points. **(A)** Mean values of the continuous ratings were significantly higher for the university band for non-patriotic pieces, but were higher for the army band for patriotic pieces. **(B)** Maximum values of the continuous ratings were significantly higher for the university band for non-patriotic pieces, but were higher for the army band for patriotic pieces.

For the mean value, there was a significant effect of piece type, such that the patriotic pieces had a higher average rating than the non-patriotic pieces. However, this main effect was subsumed by a significant interaction between piece type and band (see Table 2 for the statistical results of this model). To further investigate the significant interaction between piece type and band, pairwise comparisons (Bonferroni corrected for multiple comparisons) indicated that, for non-patriotic pieces, the university band was rated significantly higher than the army band (*β* = −1.05, SE = 0.39, *t* = −2.68, *p* < 0.001). For patriotic pieces, the army band was rated significantly higher than the university band (*β* = 0.95, SE = 0.37, *t* = 2.56, *p* = 0.01). For the maximum (or, peak) value, there was a significant interaction between piece type and band. Pairwise comparisons indicated that, for non-patriotic pieces, the university band was rated significantly higher than the army band (*β* = −1.10, SE = 0.36, *t* = −3.06, *p* = 0.003); for patriotic pieces, the army band was rated significantly higher than the university band (*β* = 0.76, SE = 0.34, *t* = 2.23, *p* = 0.02). For the variation (SD) of overall ratings, there were no significant main effects or interactions. See Table 2 for the full results of these models and Figure 2 for a graphical depiction of the results.

**Table 2:** Fixed effects for three metrics extracted from the continuous ratings (mean, max, and variation).

Fixed effect	*β*	SE	*t*	*p*	*sig.*
Mean					
Condition	−0.13	0.47	−0.28	0.77	
Piece type	0.68	0.26	2.53	0.01	*
Band	0.05	0.27	0.18	0.85	
Condition × Piece type	0.87	0.53	1.61	0.11	
Condition × Band	0.60	0.54	1.12	0.26	
Piece Type × Band	−2.01	0.53	−3.73	<0.001	***
Condition × Type × Band	1.89	1.07	1.75	0.08	
Max					
Condition	0.17	0.52	0.33	0.74	
Piece type	0.43	0.24	0.69	0.07	
Band	0.17	0.24	0.69	0.49	
Condition × Piece type	−0.06	0.49	0.12	0.89	
Condition × Band	0.51	0.49	1.04	0.30	
Piece type × Band	−1.86	0.49	−3.78	<0.001	***
Condition × Type × Band	1.21	0.98	1.23	0.22	
Variation (SD)					
Condition	0.43	0.23	1.83	0.07	
Piece type	−0.008	0.13	0.09	0.92	
Band	0.01	0.26	−0.84	0.40	
Condition × Piece type	−0.22	0.26	−0.23	0.81	
Condition × Band	−0.06	0.26	−0.84	0.39	
Piece Type × Band	−0.22	0.26	−0.84	0.39	
Condition × Type × Band	0.57	0.52	1.09	0.27	

* *p < 0.05*

*** *p < 0.001*.

*Categorical predictors were coded as follows: Condition (lab* = *−0.5, live* = *0.5); piece type (non-patriotic* = *−0.5, patriotic* = *0.5); band (army* = *−0.5, university = 0.5)*.

## Follow-Up Experiment

Due to the nature of studying a “real world” concert experience, this experiment had several limitations. For one, participants listened to each piece in the same order; there was no possibility of counterbalancing or randomizing the order across participants. Therefore, it could be the case that the effects seen here were simply order effects – it might be that listeners simply experience more pleasure during the first and last pieces they hear, and those pieces just happened to be congruent between piece type and band. Another limitation is that participants did not hear the same exact piece for each band – that is, the patriotic piece played by the university band was not the same as the patriotic piece played by the army band. Prior to the concert, the band directors discussed their program with this study in mind and attempted to match the pieces as closely as possible. While the fact that the pieces were not identical is a limitation in terms of experimental control, it also is a natural consequence of doing this type of research in a “real world” concert scenario. Although we saw effects of congruence between band and piece on pleasure ratings, it is of course possible that listeners happened to prefer the patriotic piece that was played by the army band and the non-patriotic piece that was played by the university band more than the two other pieces. A final limitation is that piece type is likely confounded with familiarity: the two patriotic pieces are highly familiar to American listeners, while the two non-patriotic pieces are likely to be unfamiliar to most listeners. However, we would not expect this effect of familiarity to be stronger for the military band than for the university band (if anything, we may expect the opposite, since most participants were students and would have increased familiarity with the university band performers).

To address these three limitations (possible order effects, the confound between the specific pieces and the context, and potential effects of familiarity), we conducted a follow-up study. In this study, online participants (*N* = 20) listened to and rated the audio-only of the pieces used in the present experiment. The audio was taken from the video recording of the concert, so it was identical to the audio heard by the participants in the main study. To test the potential effects of order, the pieces were presented randomly. Participants listened to each piece and then rated how much pleasure they felt when listening, as well as their familiarity with the piece, each on a 10-point scale. To test for order effects, we analyzed the pleasure ratings based on order. A repeated-measures ANOVA revealed no effect of order on pleasure ratings, *F*(3,57) = 0.46, *p* = 0.71, *η_p_^2^* = 0.02 (see Supplementary Material for additional details). This suggests that the effects reported in our main study are not likely due to order effects.

To investigate the second potential limitation, we conducted identical analyses to those reported in our main study, using the data collected from our follow-up study. Importantly, participants were unaware of which band was playing which piece, eliminating any possible influence of knowing the identity of the musical artist. In this case, we found no significant main effects of the band, piece type, or their interaction (for the full details of these results see the Supplementary Material). This suggests that it was not simply that participants liked certain pieces more than others, but that these pieces were preferred in a contextually congruent context. Finally, to address the issue of familiarity, we conducted the same analyses reported in our main study using the familiarity ratings as the outcome variable (rather than the pleasure ratings). As we had expected, the participants did rate significantly higher familiarity for the patriotic pieces (*M* = 7.22, SE = 0.48) than the non-patriotic pieces (*M* = 3.02, SE = 0.48). However, there was no interaction between piece type and band, so this main effect of familiarity was not likely to influence the interaction we found in our main study reported here. That is, the patriotic piece played by the army band was equally familiar to listeners as the patriotic piece played by the university band. See the Supplementary Material for the full details regarding the methods and results for this follow up study.

## Discussion

The present study investigated the effect of viewing context and musical congruity on felt pleasure in response to music. We manipulated two contextual effects: viewing setting (live concert vs. recorded concert) and band type. To investigate these effects, we measured both discrete, overall ratings and continuous ratings during music listening. For the overall ratings, participants rated their current emotional state, feelings about the United States military, and feelings about attending military band and university band concerts. For continuous ratings, participants continuously rated their felt pleasure while listening to four pieces of music: two were traditional United States patriotic pieces, and two were non-patriotic pieces. Overall, our results indicate that participants report higher pleasure when listening to a piece of music that is congruent with the musical artist. We discuss these findings in detail, and their implications for music-making during the COVID-19 pandemic, below.

### Differences Between Live and Recorded Concerts: Implications for Music-Making During and After the COVID-19 Pandemic

In terms of the overall ratings, we found a main effect of condition (live vs. lab) on participants’ feelings towards the United States military. Overall, participants in the live group reported more positive feelings towards the United States military than those in the lab group. This could be due to selection bias, as the participants who chose to attend the live concert knew they would be seeing a military band, and perhaps already held positive feelings about the United States military. We attempted to avoid this type of bias by recruiting participants broadly throughout the university community instead of asking people who were already in the audience to participate. It may be the case that live audience members felt more positively towards the military due to a sense of connection by being in the same room as the artist; if this speculation is true, musical artists may have to work harder to build that sense of connection during virtual performances.

In terms of the continuous ratings, we found no significant differences between live and lab groups. While we expected that the live group would report overall higher pleasure than the lab group, our results did not confirm this prediction. That is, listeners in both groups rated similar levels of pleasure, and both groups showed consistent effects of musical congruency such that listeners experienced more pleasure when listening to music that was congruent with the musical artist. While we found little difference between the two groups, we do not intend to suggest that there are no additional differences between viewing a live concert and viewing a recorded version of the concert. Of course, there are many aspects of live music that cannot be recreated virtually. However, our results suggest that participants do experience pleasurable emotional responses when viewing recorded concerts – this provides support for the current trend of musical artists live streaming during the COVID-19 pandemic.

In our study, participants in the lab setting knew they were watching a pre-recorded concert (similar to watching a recorded concert video on YouTube). An interesting direction for future research would be to compare responses to a pre-recorded concert and a live-streamed concert. In that scenario, the viewing context would be the same (i.e., watching a concert alone, on a computer screen) but the “live” experience would be different. Some motivations for attending live concerts are still present even in the live-streamed version, such as the novelty and unexpectedness of seeing something happen in real-time (Brown and Knox, 2017). In this way, live-streamed concerts may be closer to an in-person experience than recorded concerts. An additional aspect of viewing a live-streamed concert, in comparison to a pre-recorded concert, is the knowledge that you are watching in real-time with other viewers. This “social” aspect of the experience may also contribute to one’s felt pleasure.

### Effects of Congruence Between Artist and Piece

The most striking result from the present work is the effect of congruence on the pleasure experienced while listening to music. Both the average and the peak of the continuous ratings were higher for congruent band-piece pairs. That is, listeners experienced more overall pleasure when listening to patriotic pieces by the army band and non-patriotic pieces by the university band. This suggests that the context in which a piece of music is heard (in this case, the band that plays the piece) can have an influence on a listener’s response to it. This expands on our prior work indicating that certain stimulus properties, such as musical genre, can influence aesthetic judgments of music (Belfi et al., 2018; Belfi, 2019). Here, we found an interaction between musical style and musical artist, which suggests that expectancy influences feelings of pleasure. It may be the case that listeners *expect* to hear patriotic music when they see a military band, and that when their expectations are confirmed they experience pleasure. There is a rich history of work suggesting that expectations are a key component of musical reward (Meyer, 1956; Huron, 2006), and recent work confirming these theories (Pearce and Wiggins, 2012; Koelsch et al., 2019). Our results do not speak to listener expectations of the musical structure itself, but rather, expectations for what type of music they will hear when attending a concert by a particular musical artist.

These results also align with other work indicating that such contextual cues have an influence on aesthetic judgments. For example, one study found that listeners reported greater enjoyment of music when they were told it was written by Mozart than when they were told it was written by a less prominent composer (Fischinger et al., 2018). Similarly, with other artforms, individuals judge artworks to be less valuable if they are duplicates of the original (Newman and Bloom, 2012), and find artworks to be more profound if they are coupled with a profound-sounding title (Turpin et al., 2019). Together, this work suggests that contextual information outside the music itself can have a strong influence on a listener’s aesthetic experience.

Additionally, our overall rating results indicated a main effect of time on listeners’ feelings towards the United States military. That is, in both the live and lab conditions, participants felt significantly more positive towards the military after seeing the military band perform. Feelings towards the military did *not* increase after viewing the university band playing a patriotic piece – this suggests that listening to United States patriotic music alone is not enough to improve feelings towards the military, but seeing a military band perform is. This finding suggests that concerts by military bands are serving their intended purpose: When military bands perform in public settings it is typically for the purposes of demonstrating the professionalism of the armed services and allowing audiences to interact with members of the military in a positive setting. Prior anecdotal evidence in the form of audience testimonials has demonstrated that military band concerts have an emotional impact on listeners; however, until the present study, there was no empirical research investigating how attitudes and perceptions may change during the concert. The Government Accountability Office in report GAO-17-657 (August 2017), recommended military bands to enhance efforts to measure the performance and effects of their activities. One of these is the relative emotional influence live music performances have on audiences, and how that influence affects their impression of the United States military. Our results suggest that military band concerts do have an overall positive effect on impressions towards the military. However, our results cannot indicate which musical pieces had the strongest influence on this effect. Future work could measure changes after each piece of a concert to get a finer-grained idea in terms of which programming most influences audience attitudes.

### Limitations and Future Directions

Our work is not without limitations. For one, despite having adequate power to detect our effects of interest, our sample size is relatively small. Additionally, participants were not randomly assigned to conditions (live vs. lab). This study, due to the nature of being a “real world” concert experience, also had several natural limitations including potential effects of order and influences of the specific pieces played. However, we conducted a follow-up experiment which suggested that these limitations may not entirely be driving our main results. While our follow-up experiment indicated no significant differences between ratings of the pieces, this null effect does not entirely rule out the possibility that certain pieces are liked more than others, regardless of congruency between piece and band. That is, while the null result was predicted and is consistent with a true congruency effect in our main experiment, the similar pattern of data between the two experiments suggests that certain pieces may be liked more than others. We suspect that there may be an interaction, such that certain pieces are liked more than others, but that this may be further bolstered by the effect of congruency with the musical artist. However, we do not have adequate power to statistically test for differences between the original and follow-up experiments, and differences in the implementation (online vs. in-person, etc.) prevent us from making direct comparisons between the two. Future work could address these types of issues up front by designing concerts specifically for experimental purposes, rather than studying a “real” concert where the experimenter has little control over the order and specific pieces played. Nevertheless, this work makes an important contribution to the ways listeners experience pleasure when viewing a naturalistic, “real world” concert.

### Conclusion

The present study investigated the effect of viewing context (live, lab) and musical congruity between artist and band on aesthetic judgments of music. We found that listeners experienced more pleasure when listening to music which was congruent with the band: listeners reported higher levels of pleasure when listening to a patriotic piece played by a military band, and a non-patriotic piece played by a university band. Overall, we found little difference in the pleasure experienced between a live and a lab setting. These results have important implications for music-making during and after the COVID-19 pandemic as artists are working to replicate the live concert experience online. Encouragingly, our results indicate that listeners can and do experience pleasure even while viewing a pre-recorded concert, suggesting that some elements of the live experience can be faithfully replicated virtually.

## Data Availability Statement

The datasets presented in this study can be found in online repositories. The names of the repository/repositories and accession number(s) can be found at: https://osf.io/c5d3h/.

## Ethics Statement

The studies involving human participants were reviewed and approved by Missouri S&T IRB. The participants provided their written informed consent to participate in this study.

## Author Contributions

AB, DS, and JC conceived of the idea and designed this study. AB, JC, and NS collected data at the live concert. NS collected the lab data. AB and NS conducted the data analysis. AB drafted the initial manuscript and the manuscript revisions. All authors collaboratively edited the manuscript and approved it for submission.

### Conflict of Interest

The authors declare that the research was conducted in the absence of any commercial or financial relationships that could be construed as a potential conflict of interest.
