# Supplementary material for: Aesthetic Judgments of Live and Recorded Music: Effects of Congruence Between Musical Artist and Piece
Source: Front Psychol. 2021 Feb 4;12:618025. doi: 10.3389/fpsyg.2021.618025 (PMC7890201; doi:10.3389/fpsyg.2021.618025)
Supplement: Supplementary file 1 [file Data_Sheet_1.docx]

**Supplemental Analyses**

To account for potential effects of participant drop-out (i.e., some participants only rated some pieces), we conducted all our main analyses a second time, only including those participants who rated all four of the pieces. This resulted in six participants in the live group (3 F, 1 M, 2 prefer not to answer; two with a family member in the military, two without, two prefer not to answer; average years of musical training = 3.79, SD=3.58; average age=18.75, SD=0.5; average years of education=14.5, SD=1.0) and ten participants in the lab group (7 M, 3 F; four with a family member in the military, six without; average years of musical training = 6.6, SD=5.12; average age = 22.16, SD=2.55; average years of education = 16.08, SD=1.67). After removing the participants who did not rate all the pieces, our main results did not substantially change (see **Table S1** and **Figure S1**).

| **Fixed effect** | **β** | **SE** | ***t*** | ***p*** | ***sig.*** |
| --- | --- | --- | --- | --- | --- |
| **Mean** |  |  |  |  |  |
| Condition | 0.34 | 0.48 | 0.69 | 0.49 |  |
| Piece Type | 0.52 | 0.32 | 1.58 | 0.11 |  |
| Band | -0.04 | 0.32 | -0.14 | 0.88 |  |
| Condition x Piece Type | 0.42 | 0.65 | 0.64 | 0.52 |  |
| Condition x Band | 0.26 | 0.65 | 0.40 | 0.68 |  |
| Piece Type x Band | -1.78 | 0.65 | -2.71 | 0.009 | ** |
| Condition x Type x Band | 2.53 | 1.31 | 1.92 | 0.06 |  |
| **Max** |  |  |  |  |  |
| Condition | 0.57 | 0.35 | 1.59 | 0.13 |  |
| Piece Type | 0.37 | 0.30 | 1.23 | 0.22 |  |
| Band | 0.19 | 0.30 | 0.66 | 0.51 |  |
| Condition x Piece Type | -0.52 | 0.60 | -0.87 | 0.38 |  |
| Condition x Band | 0.38 | 0.60 | 0.64 | 0.52 |  |
| Piece Type x Band | -1.94 | 0.60 | -3.23 | 0.002 | ** |
| Condition x Type x Band | 1.12 | 1.20 | 0.93 | 0.35 |  |
| **Variation (SD)** |  |  |  |  |  |
| Condition | 0.37 | 0.25 | 1.42 | 0.17 |  |
| Piece Type | -0.07 | 0.13 | -0.56 | 0.57 |  |
| Band | -0.02 | 0.13 | -0.16 | 0.86 |  |
| Condition x Piece Type | -0.55 | 0.27 | -2.00 | 0.05 |  |
| Condition x Band | -0.17 | 0.27 | -0.64 | 0.52 |  |
| Piece Type x Band | -0.47 | 0.27 | -1.72 | 0.09 |  |
| Condition x Type x Band | 0.11 | 0.55 | 0.21 | 0.83 |  |

**Table S1.** Fixed effects for three metrics extracted from the continuous ratings (mean, max, and variation) including only subjects who rated all four pieces.* *p*<0.05, ** *p*<0.01, *** *p*<0.001

**Supplemental Experiment 1**

In this experiment, 20 participants (4 M, 15 F, 1 nonbinary) were recruited online via Prolific (Palan & Schitter, 2018). Participants were recruited from the United States, had a 95% approval rate and had completed at least 100 tasks on Prolific. Participants listened to the four musical pieces used in the present study. To prevent participants from knowing which band was playing which piece, the participants only listened to the audio of the pieces. Pieces were also presented in a randomized order. Participants listened to each piece and rated how much pleasure they experienced while listening to the piece, as well as their familiarity with the piece, each on a 10-point scale. To ensure participants were paying attention to the task, we also included a foil question that asked participants to type a specific word into a box. None of the participants failed the foil question.

To first test order effects, we conducted a repeated-measures ANOVA to test for the effect of order on the pleasure experienced. This revealed no effect of order on pleasure ratings F(3,57)=0.46, *p*=0.71, _p_η^2^=0.02. See **Figure S1** for a graphical depiction of this result. This suggests that any results found in our main study were not simply due to order effects.


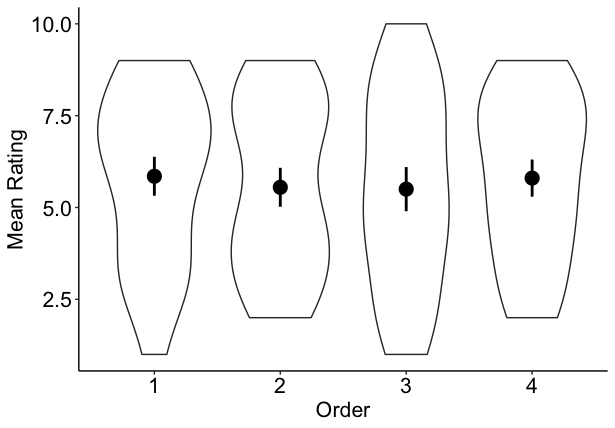


**Figure S1.** Pleasure ratings from supplemental experiment 1. Circles indicate means, error bars indicate standard error of the mean. Violin plots illustrate distributions of the individual data points. There were no significant differences between the order of presentation.

To test the potential effects of the confound between musical piece and context congruence, analyzed the data in the same manner of the main experiment. In contrast to the result from the main study, in this follow-up experiment we found no significant main effect of piece type (β=-0.05, SE=0.25, *t*=-0.19, *p*=0.84), band (β=-0.20, SE=0.25, *t*=-0.78, *p*=0.43) or interaction between band and piece type (β=-0.80, SE=0.50, *t*=-1.57, *p*=0.12). See **Figure S2** for a graphical depiction of the data. This suggests that it was not the case that participants simply preferred certain pieces over others.

**
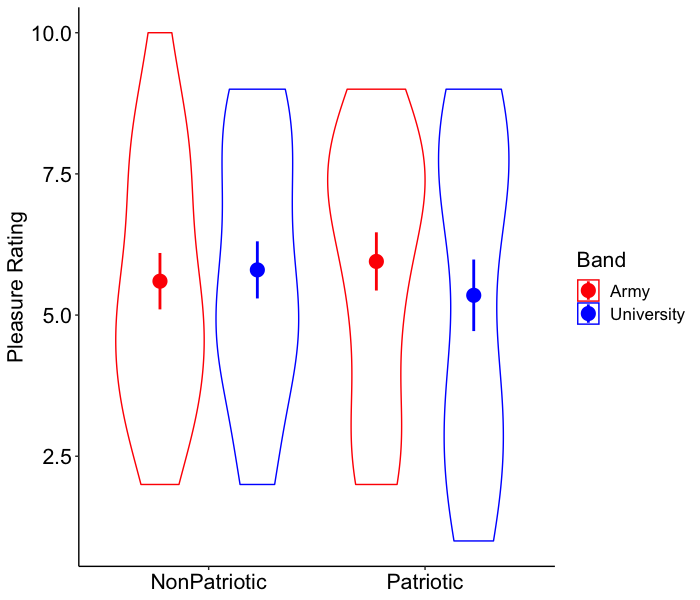
**

**Figure S2.** Pleasure ratings from Supplemental Experiment 1. Circles indicate means, error bars indicate standard error of the mean. Violin plots illustrate distributions of the individual data points. There were no significant differences between conditions.

Finally, we investigated whether familiarity varied by piece type. Our analysis revealed a significant main effect of piece type (β=4.20, SE=0.47, *t*=8.80, *p*<0.001) but no main effect of band (β=0.05, SE=0.47, *t*=0.10, *p*=0.91) or interaction between piece type and band (β=-1.0, SE=0.95, *t*=-1.04, *p*=0.29). This suggests that participants were more familiar with the patriotic than non-patriotic pieces, but it is unlikely that this interacted with the contextual congruency. See **Figure S3** for a graphical depiction of the data.


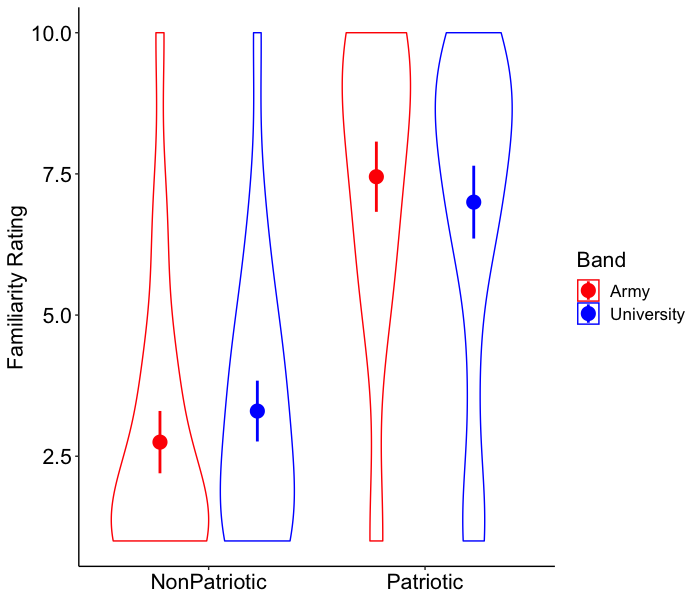


**Figure S3.** Familiarity ratings from Supplemental Experiment 1. Circles indicate means, error bars indicate standard error of the mean. Violin plots illustrate distributions of the individual data points. There was a significant main effect of piece type (patriotic pieces were significantly more familiar than non-patriotic pieces).
